# The NR-6: a new brief measure of nature relatedness

**DOI:** 10.3389/fpsyg.2013.00813

**Published:** 2013-11-01

**Authors:** Elizabeth K. Nisbet, John M. Zelenski

**Affiliations:** ^1^Department of Psychology, Trent UniversityPeterborough, ON, Canada; ^2^Department of Psychology, Carleton UniversityOttawa, ON, Canada

**Keywords:** nature relatedness, environmental attitudes, sustainable behavior, subjective well-being, happiness, scale development

## Abstract

The construct of (dis)connection with nature or “nature relatedness” has become increasingly useful in the study of environmental behavior as well as psychological health and well-being. Strong nature relatedness is associated with greater happiness and ecologically sustainable behavior. A number of scales reliably assess individual differences in nature relatedness, but some circumstances may necessitate a brief measure. We developed a short-form version of the nature relatedness scale (NR-6), comprised of 6 items from the “self” and “experience” dimensions, and tested the new scale's predictive ability across multiple samples and with longitudinal data in students, community members, and business people. The new NR-6 scale demonstrated good internal consistency, temporal stability, and predicted happiness, environmental concern, and nature contact. This new brief measure of connectedness may have advantages where time and space are limited and the research context requires an assessment of connectedness elements rather than environmental attitudes.

## Introduction

As environmental problems worsen, researchers are directing their attention toward human-nature relationships and their effects on environmentally sustainable behavior. The construct of nature relatedness (NR; and the self-report scale by the same name) captures individual differences in the way people view their relationship with the natural world (Nisbet et al., 2009). High nature relatedness, or a strong subjective connection with nature, is typically associated with greater happiness and environmental concern. Disconnection likely has harmful consequences for both human and environmental health, yet is a regular consequence of the modern lifestyles that often separate people (physically and psychologically) from the natural world. Thus, research on nature relatedness has potentially important implications, and the NR scale is increasingly used in research on sustainability and well-being. As these research contexts expand, the scale's length at 21 items can sometimes pose a problem. Here, we describe the development and validation of a new short version of the nature relatedness scale.

The theoretical background of nature relatedness draws on Wilson's (1984) biophilia hypothesis. He argued that because humans evolved in nature, we developed an innate need to connect with all life; other living things supported our health and survival. The biophilia hypothesis helps to explain our connection (and the consequences of disconnection) with the natural world. Kellert and Wilson (1993) suggest that the learning and appreciation of biodiversity has likely been embedded in our biology, and that nature is essential for our health and development. The biophilia hypothesis has inspired a wide variety of research, for example, on landscape preference and phobias (Kaplan and Kaplan, 1989; Heerwagen and Orians, 1993; Ulrich, 1993). The popularity of outdoor wilderness activities, gardening, our relationship with animals, and our fondness for natural scenery are also evidence of biophilia (Lawrence, 1993; see Kahn, 1999, for a review). The mood (MacKerron and Mourato, in press), cognitive (Berman et al., 2008), health (Frumkin, 2001), and longevity (Mitchell and Popham, 2008) benefits associated with proximity to greenspace are also indicative of nature's importance for optimal health and well-being (see also the broad review by Selhub and Logan, 2012).

Despite these benefits, and people's evident attraction to nature, there are individual differences in how people connect with nature. Indeed, people relate to the physical environment differently, and these environmental dispositions are relatively stable and trait-like McKechnie (1977). Moreover, objective contact with nature is not fully equivalent to the subjective sense of connection that likely fosters sustainable attitudes and well-being. Some people may be very connected to their local ecosystems, while others may view themselves as completely separate from the natural environment. Urban-dwelling people, in particular, may have little or no contact with nature (Maller et al., 2005). People likely find it difficult to value and care for the environment if they feel separated from nature and it is not part of their experience. Individual differences in how connected people are with nature may reflect how aware they are of biophilia or how much their biophilic tendencies are supported or suppressed. Many people may have lost their connection to the natural world (Conn, 1998), and these damaged human-nature relationships may be contributing to environmentally destructive behavior as well as unhappiness.

Schultz (2000) argues that environmental concerns are directly related to the degree with which people see themselves as part of nature. In other words, if people do not value nature or care about the environment they are not likely to protect it (Howard, 1997). The more connected people are to nature, the more they will be aware of their own actions and be concerned for all living things (Schultz, 2000). This type of “biospheric” attitude reflects a strong human-nature connection, whereas exclusive concerns for one's self (“egoistic” concerns) indicate a damaged relationship. Nature relatedness and biospheric concern are associated with less self-interest and more consideration of the larger environment, in other words, more environmental concern (Schultz, 2000; Mayer and Frantz, 2004; Dutcher et al., 2007) and self-reported environmental behavior (Schultz, 2001, 2002; Clayton, 2003; Nisbet et al., 2009).

With continuing environmental destruction, loss of biodiversity, and species extinction, we may be losing many of the elements necessary to trigger and nurture our biophilia (Thomashow, 1998). A damaged environment is unlikely to extinguish our need to connect with nature, however, it may diminish our appreciation for the role of natural diversity in healthy physical and psychological development, and reduce the opportunity for future generations to benefit from nature (Kellert, 1997). The fact that our biophilic tendencies have not resulted in more widespread environmental behavior suggests value in further studying individual differences in levels of connectedness.

To that end, a number of assessment tools have been developed to capture subjective connectedness with nature (e.g., nature relatedness, connectedness to nature, connectivity with nature, environmental identity; Schultz, 2000; Clayton, 2003; Mayer and Frantz, 2004; Dutcher et al., 2007; Nisbet et al., 2009). It is possible to make small distinctions among these constructs and assessment tools, but research to date suggests considerable similarity (Tam, 2013). Here we focus on the particular example of nature relatedness.

Nisbet et al. (2009) proposed the construct of nature relatedness to capture several facets of human-nature relationships—cognition, affect, and experience—and to measure people's interest in, fascination with, and desire for nature contact. Nature relatedness is similar to the notion of an ecological identity (a sense of self that includes nature), but is a broader concept encompassing emotions, experiences, and an understanding of human interconnectedness with all other living things. Nature relatedness is not simply a love of nature, or enjoyment of only the superficially pleasing facets of nature, but rather an awareness and understanding of all aspects of the natural world, even those that are not aesthetically appealing or useful to humans. Nature relatedness may be indicative of how much an innate need to connect with nature (biophilia) has (or has not) been nurtured. Considering the biophilia hypothesis, it also follows that a strong sense of nature relatedness should predict happiness and well-being more broadly.

Psychologists conceptualize happiness, or subjective well-being, in a variety of ways. One approach to defining subjective well-being—a hedonic approach—is to focus on the quantity of positive and negative emotions, and satisfaction with one's life (Diener, 2000). Another approach, from a humanistic perspective, uses the term psychological well-being and includes other adaptive characteristics such as sense of purpose and meaning in life (Ryff and Keyes, 1995). Drawing on both traditions, research on nature relatedness has considered happiness as a multidimensional construct and has assessed links with hedonic and eudaimonic indicators. For example, people higher in trait nature relatedness report more life satisfaction, vitality, and positive affect, as well as greater purpose in life, autonomy, and personal growth (Howell et al., 2011; Nisbet et al., 2011; Tam, 2013). The association between happiness and nature relatedness remains (or, in some cases, becomes stronger) after controlling for environmental attitude measures. Controlling for other subjective connections (e.g., with family, culture, group identities) also does not remove the link between nature relatedness and happiness (Zelenski and Nisbet, in press). Thus, it appears that nature relatedness is distinct from both environmental attitudes and a general sense of connection. There is something special about how people view their relationship with nature.

The construct of nature relatedness has been useful in understanding individual differences in environmental behavior and well-being. As interest in the construct has grown, it has also become clear that the scale's length of 21 items makes it too unwieldy for some research contexts. We therefore undertook an effort to develop a short version that was similar to the original (e.g., in terms of its content and correlates) and retained good psychometric properties. Two similar brief measures currently exist. Schultz's (2001) inclusion of nature in self (INS) is a single item, and Dutcher et al.'s (2007) connectivity with nature is five items (that includes the single INS item). (Other similar scales have >10 items). Although these measures likely provide reasonable substitutes, we note that a recent comparison found them to be somewhat less reliable, less strongly related, and somewhat distinct in predicting outcomes, compared to longer scales (Tam, 2013). Thus, there appears to be potential in a new abbreviated scoring of the nature relatedness scale.

To create the short version, we selected items from the 21-item scale that were representative of the theoretical foundations of the nature relatedness construct. Drawing on data from over 1200 previous participants, we examined frequency distributions to find items that discriminate low from highly nature related people well, and looked for items that had relatively normal distributions. We also examined individual items' correlations with other conceptually related scales that assessed environmental attitudes and subjective well-being. Using these criteria, we selected six nature relatedness items that performed very similarly to the full 21-item scale. Four of the items assess self-identification with nature, a sense of connectedness that may be reflected in spirituality, awareness or subjective knowledge about the environment, and feelings of oneness with nature: “I always think about how my actions affect the environment,” “My connection to nature and the environment is a part of my spirituality,” “My relationship to nature is an important part of who I am,” and “I feel very connected to all living things and the earth.” Two additional items capture individual differences in the need for nature and comfort with wilderness, as well as awareness of local wildlife or nearby nature: “My ideal vacation spot would be a remote, wilderness area” and “I take notice of wildlife wherever I am” (Appendix A).

It is worth noting that the six selected items represent only two of three factors observed in an exploratory factor analysis of the full scale (Nisbet et al., 2009). Those subscales were interpreted as a sense of identification (“self”), contact with nature (“experience”), and pro-nature conservation attitudes (“perspective”). Although initially hesitant to omit all perspective items, we ultimately concluded that this was the best choice for a few reasons. Perhaps obviously, our selection was done relatively blind to the subscales, i.e., we followed reasonable criteria without regard to them. The data strongly informed which items were selected, and it is indeed telling that validity correlations, particularly those with environmental attitudes, were strong without the pro-conservation items. At a conceptual level, identification and actual connection with nature seem more central to the construct of nature relatedness, and it seems fitting that these subscales form the essential items of a short version. It may be that the “perspective” subscale assesses something that is less nature relatedness and more the related, but distinct, construct of pro-environmental attitudes.

We present data from four studies that assessed the links among nature relatedness, environmental attitudes, and subjective well-being. The first three of these draw on archival findings, comparing the new short scoring to the full scale in the data we used to initially validate the full scale and more recently used to select the short scale items. We then present new data, i.e., collected after the new scoring was determined, that replicates and further validates the short version of the scale. Across studies, we expected the NR-6 measure to correlate positively with happiness and environmental behavior. We also anticipated that the short-form NR scale would predict the amount of time people spend in contact with nature.

## Study 1

### Method

#### Participants and procedure

One hundred and eighty-four undergraduate students from the psychology participant pool were recruited for a study on “personality and well-being” (reported previously as Study 1, phase 2 in Nisbet et al., 2009, and Study 1 in Nisbet et al., 2011). Most participants (82.1%) were first year students and the majority were female (67.4%; *n* = 124; *n* = 60 males). The average age was 19.48 years (*SD* = 2.83). Participants completed pen and paper versions of the nature relatedness scale, well-being and environmental measures in the laboratory, in exchange for course credit.

#### Materials

The 21-item *Nature Relatedness Scale (NR)* assesses subjective connectedness with the natural environment. Participants respond to statements using a 5-point Likert scale (1 = *strongly disagree*, 5 = *strongly agree*) and items are averaged with higher scores indicating stronger connectedness. The new *short-form Nature Relatedness Scale (NR-6)* was computed by averaging the appropriate items. Reliability statistics for all scales are in Table 1, in Results (see Appendix B for the means and standard deviations for all studies).

**Table 1:** **Study 1—nature relatedness correlations with well-being and environmental measures in students (*n* = 184)**.

	**NR-6 (.83)**	**NR-21 (.87)**
NR mean (*SD*)	3.00 (.86)	3.28 (.60)
Positive affect (0.84)	0.27^**^	0.29^**^
Negative affect (0.86)	−0.06	−0.11
Satisfaction with life (0.84)	0.04	0.13^†^
Autonomy (0.78)	0.23^**^	0.28^**^
Personal growth (0.82)	0.22^**^	0.29^**^
Purpose in life (0.76)	0.11	0.19^*^
Environmental mastery (0.77)	0.01	0.09
Self-acceptance (0.90)	0.12	0.18^*^
Positive relations with others (0.84)	0.00	0.10
Ecology scale		
Verbal commitment (0.73)	0.56^**^	0.53^**^
Actual commitment (0.72)	0.41^**^	0.42^**^
Affect (0.79)	0.54^**^	0.57^**^
New ecological paradigm (0.75)	0.38^**^	0.54^**^
New ecological consciousness (0.83)	0.53^**^	0.60^**^

† *p < 0.10*,

* p < 0.05,

** p < 0.01. Cronbach's alpha in parentheses. Average absolute value difference between NR-6 and NR-21 correlations: r = 0.073 (using Fischer's z transformations).

***Happiness indicators.*** The *Positive Affect Negative Affect Schedule (PANAS*, Watson et al., 1988) was used to measure trait positive and negative affect. Participants indicated how much, in general, they felt each of 20 emotions, using a 5-point Likert scale ranging from 1 (*very slightly or not at all*) to 5 (*extremely*). Responses to the 10 positive and 10 negative emotion words were averaged, separately, to create positive and negative affect scores.

The *Psychological Well-Being Inventory* (Ryff, 1989) assessed six dimensions of eudaimonic well-being: autonomy, environmental mastery, positive relations with others, self-acceptance, purpose in life, and personal growth. Respondents were asked about 54 statements (9 for each dimension) pertaining to various aspects of their lives, using a Likert scale ranging from 1 (*strongly disagree*) to 6 (*strongly agree*) and items from the respective dimensions were averaged to a create score for each of the six dimensions.

The *Satisfaction With Life Scale* (Diener et al., 1985) asked participants to respond to five statements concerning their life satisfaction on a Likert scale ranging from 1 (*strong disagreement*) to 6 (*strong agreement*). Items were averaged to produce a life satisfaction score.

Three subscales of the *Ecology Scale, Short-Form* (Maloney et al., 1975) assessed *verbal* and *actual commitment* and *affect* (10 items each) toward ecological issues concerning transportation, monetary donations, consumer purchases, pollution, political activism, and general awareness in a “true” or “false” response format. The respective items were summed to create overall scores for verbal commitment and actual commitment and affect.

The *New Ecological Paradigm Scale* (Dunlap et al., 2000) assessed ecological worldview. Participants rate 15 statements on a 5-point Likert scale ranging from 1 (*strongly agree*) to 5 (*strongly disagree*). Unidimensional scoring was used, averaging items such that higher scores indicate stronger environmental views.

The *New Ecological Consciousness Scale* (Ellis and Thompson, 1997) assesses environmentalism. Participants rate 10 items about ecological crises, overpopulation, and human responsibility for environmental degradation using a 7-point Likert scale ranging from 1 (*strongly agree*) to 7 (*strongly disagree*). Items were averaged for scoring with higher values indicating stronger environmentalism.

### Results

To test the predictive ability of the new short-form nature relatedness scale, we correlated the NR-6 and full 21-item NR scale with the well-being and environmental variables. We also calculated the average absolute difference between NR-6 and NR-21 correlations with the outcome variables (using Fischer's *r* to *z* transformations). The short-form scale showed a similar pattern of correlations as the full scale, with a few exceptions (Table 1). Relationships with satisfaction with life, self-acceptance, and purpose in life were only significant for the full NR scale (the NR-6 correlations were in the expected direction, however). The short-form and full NR scales were strongly correlated (*r* = 0.90, *p* < 0.01).

## Study 2

### Method

#### Participants and procedure

Data from 145 Canadian middle managers (87 men, 56 women; 2 did not indicate sex) was collected as part of a study on personality and work/life balance (reported as Study 2 in Nisbet et al., 2009, 2011). Respondents were generally middle-aged (*M* = 42.37 years, *SD* = 8.80, range: 24–70). Participants completed questionnaires online assessing nature relatedness and well-being, and then reported twice weekly for eight weeks on various work and home life activities, including how often they spent time outdoors and in nature.

#### Materials

Similar scales to Study 1 were administered online: the 21-item *Nature Relatedness Scale* (we also computed scores for the short form NR-6), the *Positive and Negative Affect Scales* (*PANAS*), the *Satisfaction with Life Scale*, and the six dimensions of the *Psychological Well-Being Inventory*. The experience sampling questions inquired about the frequency of time spent “outdoors” and “in nature (e.g., the bush)” over the previous three days, using a 5-point Likert scale (0 = *not at all*, 4 = 7 *or more times*). The twice-weekly experience sampling reports (854, in total) were aggregated to create an indicator of average outdoor and nature contact for each participant.

### Results

As in Study 1, positive affect and personal growth correlated with the new NR-6 scale although other well-being measures such as autonomy did not (Table 2). The NR-6 correlated positively with both the frequency of time outdoors and time in nature. The short-form scale also correlated highly with the full 21-item scale (*r* = 0.88, *p* < 0.01).

**Table 2:** **Study 2—nature relatedness correlations with well-being and nature contact measures in middle managers (*N* = 145)**.

	**NR-6 (0.84)**	**NR-21 (0.87)**
NR mean (*SD*)	3.39 (0.85)	3.67 (0.58)
Positive affect (0.83)	0.25^**^	0.23^**^
Negative affect (0.87)	−0.01	0.05
Satisfaction with life (0.88)	0.09	0.00
Autonomy (0.69)	0.11	0.16^†^
Personal growth (0.75)	0.16^†^	0.19^*^
Purpose in life (0.87)	0.10	0.12
Environmental mastery (0.80)	−0.05	−0.00
Self-acceptance (0.83)	0.06	0.02
Positive relations with others (0.78)	0.08	0.07
Outdoor frequency	0.24^*^	0.25^**^
Nature frequency	0.26^**^	0.30^**^

† p < 0.10,

* p < 0.05,

** p < 0.01. Cronbach's alpha in parentheses. Average absolute value difference between NR-6 and NR-21 correlations: r = 0.039 (using Fischer's z transformations).

## Study 3

### Method

#### Participants and procedure

Undergraduate students enrolled in psychology, biology, geography, and natural history courses were recruited for a study on environmental views and completed nature relatedness, well-being, and environmental scales at the beginning of the school term (findings on the 21-item NR scale in relation to well-being, in a subset of participants, are reported as Study 3 in Nisbet et al., 2011). Most students (82.5%) were in their first year of university. More women (59.9%, *n* = 212) than men (*n* = 142) participated and the mean age was 20.03 (*SD* = 4.36). Questionnaires were completed in classrooms as part of a larger study on environmental education. Psychology students received course credit and other participants were entered in a draw for a $200 cash prize.

#### Materials

Participants completed many of the same scales used in Studies 1 and 2: the 21-item *Nature Relatedness Scale* (and, again, we calculated the short form NR-6), the *PANAS*, the *Satisfaction with Life Scale*, and three of the six *Psychological Well-Being* dimensions (autonomy, personal growth, purpose in life). We included the *Vitality Scale* (Ryan and Frederick, 1997) to capture feelings of energy and being alive. Six items about vitality are rated on a 7-point Likert scale ranging from 1 (*not at all true*) to 7 (*very true*) and averaged to calculate scores. Reliability statistics for all measures are included in Table 3, in Results.

**Table 3:** **Study 3—nature relatedness correlations with well-being and environmental measures in students (*N* = 354)**.

	**NR-6 (0.86)**	**NR-21 (0.90)**
NR mean (*SD*)	3.34 (0.96)	3.65 (0.68)
Positive affect (0.83)	0.21^**^	0.25^**^
Negative affect (0.82)	−0.06	−0.08
Vitality (0.83)	0.21^**^	0.25^**^
Autonomy (0.78)	0.21^**^	0.25^**^
Personal growth (0.74)	0.29^**^	0.36^**^
Purpose in life (0.79)	0.13^*^	0.19^**^
Ecology scale		
Verbal commitment (0.72)	0.64^**^	0.70^**^
Actual commitment (0.74)	0.55^**^	0.57^**^
Affect (0.77)	0.57^**^	0.63^**^
Environmental concern		
Egoistic (0.88)	−0.00	0.00
Altruistic (0.89)	0.10^†^	0.11^*^
Biospheric (0.92)	0.43^**^	0.50^**^
Sustainability—attitudes (0.51)	0.55^**^	0.59^**^
Sustainability—behavior (0.68)	0.53^**^	0.63^**^
Inclusion with nature in self	0.68^**^	0.69^**^

† p < 0.10,

* p < 0.05,

** p < 0.01. Cronbach's alpha in parentheses. Average absolute value difference between NR-6 and NR-21 correlations: r = 0.056 (using Fischer's z transformations).

Participants completed the same three *Ecology subscales* (verbal and actual commitment, and affect) used in Studies 1 and 2. Several other measures were included as part of the study on environmental education.

The *Inclusion of Nature In Self* S*cale* (Schultz, 2002) assesses participants' feelings of closeness to the natural world. This single item measure consists of seven pairs of circles, each with labels (“me” and “nature”), and with varying degrees of overlap. Participants choose which image best represents their inclusion with nature (image1 being least inclusive and 7 being the most inclusive).

*Environmental Concern* (Schultz, 2000) evaluates the structure of participants' concern for the environment. The scale differentiates between three types of motivation or concern: egoistic (concern for environmental effects on one's own well-being); altruistic (concern based on the environmental effects on other humans); and biospheric (concern for the impact of environmental problems on all other living things). Participants indicate their concern (1 = *not important* to 7 = *supreme importance*) for the environment due to the consequences for 12 items (various human and non-human animals). Items within each subscale are averaged to create biospheric, altruistic, and egoistic scores for each participant.

A *Sustainability Survey* was added to ask specific questions about participants' attitudes concerning sustainability, as well as self-reported transportation, recycling, activism and purchasing patterns. The sustainability attitude items were averaged to create an attitude score and the number of activities reported by each participant was summed to create a sustainable behavior score.

### Results

We correlated the new NR-6 scale and the full NR scale with the well-being and environmental measures. The NR-6 correlated with all of the environmental measures and most of the well-being indicators. The new short NR scale was strongly correlated with the full scale (*r* = 0.91, *p* < 0.01).

## Study 4

### Method

The pattern of findings from the data we revisited in Studies 1, 2, and 3 suggested the short NR scale, similar to the full version, could be useful in predicting differences in well-being, environmental attitudes and behavior, as well as actual nature contact. In a new, longitudinal study, we tested the temporal stability and predictive validity of the NR-6 in an online study with more diverse participants. (The baseline correlations between NR and well-being use a subset of the sample reported in Zelenski and Nisbet, in press, Study 1, as part of a separate study).

#### Participants and procedure

Two hundred and seven community and student participants completed questionnaires online as part of a larger study about mindful awareness and happiness (748 people began the study (filled out an initial baseline survey), however, only about one quarter (*N* = 207) of those continued on and completed the month of surveys). Participants provided informed consent and demographics information on the study website and agreed to receive email notices for several web-based surveys assessing awareness of their surroundings and happiness. The website program generated automated emails for surveys twice per week over one month, and a final follow-up survey at the end of the month. (A six-month follow up survey was also sent to participants but low response rates precluded statistical analyses). Each participant received a link and secure password to complete the appropriate online questionnaires. Participants completed questionnaires measuring happiness and connection with nature at the beginning and end of the study. All surveys included questions about participants' time use over the previous three days, to assess regular nature contact. The final survey included additional measures of environmental concern, and behavior.

As part of the larger study, all participants completed brief guided writing exercises about their environment (Nisbet, 2011). Participants were randomly assigned at the beginning of the study to a condition (indoor vs. outdoor environment writing) and were given prompts at the end of each survey to write about thoughts and experiences (e.g., related to food, music, favorite indoor and outdoor places). The experimental conditions had no impact on the outcome variables in the present study so results reported are for all participants, collapsed across conditions.

Community participants (*n* = 84) were recruited using advertisements on Facebook, Google, Craigslist, and websites that list web-based experiments. The mean age of community respondents was 37.86 (*SD* = 15.01; range: 16–72) and the majority were women (*n* = 66; 78.6%). Community participants were mostly Caucasian (*n* = 67; 82.7%); 6.2% were Asian, and 2.5% were Black. Participants were Canadians (*n* = 20, 23.8%), New Zealanders (*n* = 30, 37.5%), and Americans (*n* = 23, 27.4%), and 28.6% had completed an undergraduate degree. More than half (60.2%) were employed and the majority were urban dwellers (72.7%), residing in the center or suburbs of a city.

Student participants (*n* = 123) were also mostly women (*n* = 95; 77.2%) attending a Canadian university and in their first year of studies. The mean age was 20.95 (*SD* = 5.60, range: 17–56). The student sample was slightly more ethnically diverse; most participants were Caucasian (*n* = 84, 70.0%); 11.7% were Asian, 4.2% were Black. Most students (89.4%) were living in the center or suburbs of a city.

Community participants were entered into a draw for $500 U.S. in exchange for completing each of the surveys. Students completed the surveys in exchange for grade-raising experimental credits in introductory psychology classes. Participants received debriefing information at the end of the study.

#### Materials

The baseline questionnaire included the new *short-form Nature Relatedness Scale (NR-6)*, however, the six statements were inserted among personality items (the *Big Five Factor Inventory*; John and Srivastava, 1999), to unobtrusively assess individual subjective connection with nature, while avoiding demand characteristics^1^. The final survey included the full 21-item nature relatedness scale. Scores were computed for the full scale (community/students α = 0.89/0.90) as well as the short-form version (community/students α = 0.90/0.89 at baseline, α = 0.89/0.90 at one month). Reliability statistics for all other measures (α) are reported in Tables 5, 6, and 7, in Results.

To test the concurrent validity of the NR-6 measure, participants also completed the *Inclusion of Nature In Self Scale* (Schultz, 2002) used in Study 3. The *INS* was imbedded in six foils that replaced nature with other concepts (e.g., family, music, culture).

We included the same *PANAS* as in previous studies. We also created an *ad-hoc* “fascination” scale with three emotion words (*in awe, fascinated, curious*) particularly relevant to nature experiences and incorporated these into the *PANAS* scales. The fascination variable was calculated by averaging scores on the three emotion words intended to capture the restorative “soft fascination” evoked by nature (described by Kaplan, 1995).

Participants completed the *Vitality Scale* and the autonomy, purpose in life, and personal growth subscales used in Study 3, the *Satisfaction with Life Scale* (from Studies 1 and 2), along with additional happiness measures.

The *Subjective Happiness Scale* (Lyubomirsky and Lepper, 1999) assessed general happiness levels with four statements or questions rated on 7-point scales of agreement. For example, “In general, I consider myself… 1 (*not a very happy person*) 7 (*a very happy person*).” Items were averaged with higher scores indicating greater happiness.

Depression symptoms were assessed using the *Center for Epidemiological Studies Depression Scale* (Radloff, 1977). This scale is designed to measure depressive symptoms in the general population and consists of 20 items such as “I did not feel like eating; my appetite was poor,” and “I felt that everything I did was an effort.” Participants rated how much of the time they felt each item during the prior week using a 4-point Likert scale ranging from 0 (*rarely*) to 3 (*most*). Scores were calculated by summing the items such that a higher overall score (maximum possible is 80) indicates greater depression.

*Nature Contact* was evaluated twice each week during the study with questions asking about daily activities and life settings (the list of activities was adapted from Kahneman et al., 2004) such as how many hours were spent, during the previous three days: “on a walk, hike, or activity in nature.” Various distractor items (e.g., “shopping,” “out at a restaurant,” “commuting to work/school,” “sending e-mail/surfing the internet”) were inserted to reduce demand characteristics. The seven reports on nature time were averaged to create aggregated scores indicative of nature contact.

After a month, participants repeated the same measures of affect, vitality, psychological well-being, and happiness administered at the beginning of the study, along with measures of nature relatedness (NR-6, NR-21), inclusion, environmental concern and behavior. The last survey also included the same ecological *verbal* and *actual* commitment subscales used in Studies 1 and 3, and the *Environmental Concern Scale*, sustainability attitudes and behavior scales used in Study 3.

### Results

Although results from studies 1, 2, and 3 suggested the new NR-6 had internal consistency, we further explored this with factor analysis in the new data sample. (Results were the same for the student and community participants, so results are reported for the entire participant sample). A maximum likelihood factor analysis (with a Promax rotation, κ = 4, to allow for overlap among potential dimensions) was conducted with the 6 items in the short form scale. All communalities were > 2.6 and a single factor was extracted (eigenvalues were 3.75, 0.733, 0.543, 0.450, 0.290, 0.234) explaining 62.48% of the total variance.

Temporal stability of the new NR-6 measure was examined by correlating baseline scores with scores on the same measure after one month. Test-retest correlations for the NR-6 indicated stability over time (i.e., correlations were significant and large; see Table 4). The short-form NR-6 scale also correlated highly with the full 21-item scale.

**Table 4:** **Study 4—test-retest correlations—baseline to one month for nature relatedness 6-item and 21-item scales**.

	**1**	**2**	**3**
1. NR-6 baseline	–	0.84^**^	0.80^**^
2. NR-6 one month	0.83^**^	–	0.93^**^
3. NR-21 one month	0.79^**^	0.91^**^	–

** p < 0.01. Community correlations are above the diagonal. Student correlations are below the diagonal.

To test the whether the short form nature relatedness scale predicted happiness, we examined correlations between the well-being indicators and the NR-6 at baseline, and the NR-6 and full NR scale at one month, in both the student and community samples (Tables 5 and 6). Additionally, we computed lagged correlations, between the NR-6 at baseline, and well-being one month later (Table 6). In general, for both community and student participants, nature relatedness was associated with higher levels of well-being. Nature relatedness at baseline (assessed with the new scale) predicted well-being a month later. The one-month correlations showed a similar pattern. The new NR-6 scale correlated positively with hedonic and eudaimonic happiness indicators, similarly to the full 21-item scale.

**Table 5:** **Study 4—nature relatedness correlations with well-being at baseline**.

	**Community (*n* = 84)**	**Students (*n* = 123)**
	**NR-6 baseline**	**NR-6 baseline**
Fascination (0.79/0.69)	0.36^**^	0.22^*^
Positive affect (0.89/0.90)	0.38^**^	0.26^**^
Negative affect (0.89/0.87)	−0.26^*^	−0.07
Vitality (0.93/0.90)	0.24^*^	0.13
Subjective happiness (0.93/0.90)	0.19^†^	0.09
Satisfaction with life (0.91/0.88)	0.09	0.14
Depression (0.72/0.70)	−0.08	−0.10
Autonomy (0.82/0.80)	0.38^**^	0.33^**^
Personal growth (0.83/0.80)	0.41^**^	0.42^**^
Purpose in life (0.78/0.81)	0.29^**^	0.18^*^

† p < 0.10,

* p < 0.05,

** p < 0.01. Cronbach's alpha (community/students) in parentheses.

**Table 6:** **Study 4—nature relatedness correlations at baseline and one month with well-being at one month**.

**Well-being 1 month**	**Community (*n* = 84)**			**Students (*n* = 123)**		
	**NR-6 baseline**	**NR-6 1 month**	**NR-21 1 month**	**NR-6 baseline**	**NR-6 1 month**	**NR-21 1 month**
Fascination (0.82/0.77)	0.08	0.14	0.11	0.08	0.08	0.11
Positive affect (0.94/0.91)	0.31^**^	0.36^**^	0.42^**^	0.22^*^	0.23^*^	0.29^**^
Negative affect (0.91/0.90)	−0.11	−0.15	−0.27^*^	−0.21^*^	−0.13	−0.19^*^
Vitality (0.96/0.94)	0.21^†^	0.27^†^	0.35^**^	0.10	0.16^†^	0.24^**^
Subjective happiness (0.92/0.87)	0.15	0.24^*^	0.27^*^	0.06	0.02	0.19^*^
Satisfaction with life (0.93/0.91)	−0.01	0.05	0.10	0.01	0.02	0.13
Depression (0.80/0.73)	0.01	−0.00	−0.09	−0.03	−0.03	−0.09
Autonomy (0.83/0.83)	0.31^**^	0.38^**^	0.45^**^	0.31^**^	0.23^*^	0.36^**^
Personal growth (0.82/0.85)	0.36^**^	0.45^**^	0.49^**^	0.37^**^	0.31^**^	0.51^**^
Purpose in life (0.84/0.79)	0.22^*^	0.23^*^	0.26^*^	0.18^*^	0.14	0.26^**^

† p < 0.10,

* p < 0.05,

** p < 0.01. Cronbach's alpha (community/students) in parentheses. Average absolute value difference between NR-6 and NR-21 correlations at 1 Month: r = 0.066 for community, r = 0.109 for students (using Fischer's z transformations).

The new nature relatedness scale was highly correlated with another measure of connectedness—the INS. Nature relatedness was also positively associated with nature contact (more so in the community sample), and was generally associated with greater environmental concern and self-reported environmental action in both student and community participants. Baseline levels of nature relatedness were positively correlated with all environmental outcome variables measured a month later (lagged and simple correlations are presented in Table 7).

**Table 7:** **Study 4—nature relatedness correlations at baseline and one month with environmental measures at one month**.

	**Community (*n* = 84)**			**Students (*n* = 123)**		
	**NR-6 baseline**	**NR-6 1 month**	**NR-21 1 month**	**NR-6 baseline**	**NR-6 1 month**	**NR-21 1 month**
Inclusion with nature in self	0.64^**^	0.74^**^	0.71^**^	0.70^**^	0.75^**^	0.69^**^
Aggregated nature contact	0.25^*^	0.36^**^	0.38^**^	0.16^†^	0.17^†^	0.22^*^
Verbal ecological commitment (0.65/0.61)	0.42^**^	0.57^**^	0.64^**^	0.42^**^	0.52^**^	0.59^**^
Actual ecological commitment (0.80/0.68)	0.45^**^	0.55^**^	0.61^**^	0.50^**^	0.59^**^	0.58^**^
Egoistic concern (0.93/0.88)	0.09	0.15	0.17	0.13	0.10	0.19^*^
Altruistic concern (0.91/0.89)	0.28^*^	0.32^**^	0.32^**^	0.25^**^	0.19^*^	0.31^**^
Biospheric concern (0.95/0.92)	0.50^**^	0.57^**^	0.59^**^	0.46^**^	0.41^**^	0.53^**^
Sustainable behavior (0.74/0.71)	0.36^**^	0.48^**^	0.55^**^	0.37^**^	0.50^**^	0.50^**^

† p < 0.10,

* p < 0.05,

** p < 0.01. Cronbach's alpha (community/students) in parentheses. Average absolute value difference between NR-6 and NR-21 correlations at 1 Month: r = 0.054 for community, r = 0.083 for students (using Fischer's z transformations).

### Discussion

Our goal was to test the reliability and predictive validity of a new brief measure of nature relatedness by comparing the brief scale's performance with that of the 21-item version. We reduced the full scale to less than a third but maintained sufficient reliability and validity. The NR-6 scale showed temporal stability, and predicted happiness and environmental outcomes, similarly to the full scale. Convergent validity with the INS ranged from 0.64 to 0.75, similar to the full scale. Overall, the six nature relatedness items (Appendix) combined to provide a reliable assessment of individual differences in nature relatedness in that the new short form measure demonstrated statistical reliability without compromising the construct validity.

Across samples, the new measure correlated with positive affect as well as eudaimonic indicators of well-being such as personal growth, purpose in life, and autonomy. Consistent with past findings (cf. Nisbet et al., 2011; Zelenski and Nisbet, in press), nature relatedness was less related to cognitive indicators of happiness (e.g., life satisfaction). The short-form measure of nature relatedness appears to capture elements of the human-nature bond that are linked to well-being similarly to the full 21-item scale, albeit with somewhat weaker relationships.

The new measure was also consistently related to environmental concern and behavior, in both student and community participants. People who are more nature related indicated greater intention to behave environmentally, and also seem to follow through, reporting more commitment and action. Nature relatedness was associated with concern for all living things (biospheric), as well as one's community and future generations (altruistic), but not with more selfish (egoistic) motives, in keeping with the notion of biophilia.

Being outdoors and having frequent nature contact promotes connectedness as well as positive moods. Given that positive emotions can broaden thought-action repertoires (Fredrickson, 2000), it follows that being happy may inspire more care and concern for the natural environment. Indeed, happy people may be more ecological minded (Brown and Kasser, 2005). Without evidence of causation, however, we would speculate that increasing happiness is not likely to produce widespread environmental behavior change. Rather, restoring damaged human-nature relationships and encouraging connectedness seem more likely to foster caring and protective behavior, and possibly happiness as well.

Despite the trait-like properties and high test-retest correlations with the NR-6, nature relatedness may not be completely fixed. Brief exposure to nature (or even photographs of nature) enhances mood and vitality and can promote connectedness temporarily (Mayer et al., 2009; Weinstein et al., 2009; Ryan et al., 2010). Given that the NR-6 often correlated with aggregated assessments of time in nature, repeated exposure to natural environments may help to increase or maintain connectedness. The applied challenge may lie in how to encourage more frequent nature contact and determine whether this leads to any lasting changes in nature relatedness.

### Limitations and future directions

There is some psychometric cost to using an abbreviated measure. Some reliability is lost by reducing the scale to 6 items, particularly when assessing relationships with well-being. The correlations with environmental variables were similar, but the well-being correlations were reduced by up to 0.05. Indeed, across all studies, the average differences between NR-6 and NR-21 correlations with outcome variables ranged from 0.039 to 0.109. Unlike the longer scale, the short form has no reverse-scored items and is more vulnerable to acquiescence bias. The 6-item measure has the advantage of reducing redundancy, participant fatigue or boredom, and because it can be embedded in other measures (e.g., personality scales, as in our Study 4) it may reduce demand characteristics. For high quality assessment of nature relatedness, the 21 item scale is preferable, however.

The short form nature relatedness scale appears to reliably predict behaviors and attitudes, despite the omission of any perspective items from the full scale. We are not suggesting this new instrument should replace well-established longer measures of nature relatedness, but rather offer an alternate when time and space are limited. There may be contexts where the short scale is inadequate or where dimensionality of the nature relatedness construct is of interest. Positive emotions appear to be less related to the attitudinal aspects of nature relatedness, for example (Zelenski and Nisbet, in press) and future work is needed to understand how awareness of environmental problems impacts well-being.

Because many of our studies included students, the samples were relatively homogenous and any findings are thus limited in generalizability. Advertising Study 4 as being about happiness was helpful in recruiting participants, but may have attracted less happy people, as opposed to a more representative sample. We conducted two of our studies online, providing accessibility to people who might not otherwise have opportunities to participate in research, or who are limited financially, geographically, or physically, from doing laboratory experiments. This is also problematic in that the online format may have inadvertently attracted people who prefer being inside and on the computer. Our findings are correlational, limiting conclusions about the causal role of nature relatedness in promoting well-being and environmental action. Further work is needed to determine the new scale's efficacy with broader outcome variables and beyond self-report measures of behavior. Human-nature relationships also likely vary greatly across cultures and research is needed to better understand how connectedness is perceived and experienced by indigenous and aboriginal peoples.

## Conclusion

A disconnection from the natural environment may be contributing to poor psychological health as well as environmentally destructive behavior (Kellert, 1997; Conn, 1998). By better understanding and developing ways to restore connections with nature we may be able to foster greater ecological concern and sustainable behavior (Howard, 1997; Schultz, 2000), as well as promote psychological well-being. By diagnosing the causes of disconnection and developing strategies to enhance nature relatedness we may be able to promote human well-being and sustainable behavior concurrently. Measuring individual differences in nature relatedness may be one way to do this.

Although the short from NR-6 may not capture all components of nature relatedness as broadly as the full scale, the new measure met the evaluation criteria: test-retest reliability, convergent validity, and a similar pattern of external correlates as the full scale. The benefit of the NR-6 is that it is a shorter measure and can be applied to a wider variety of research studies.

A brief assessment of nature relatedness is useful in research contexts where time or space is limited, e.g., field studies, citizen science ventures, when other tests need to be administered, or when reducing face validity is a concern. The six NR items can be embedded in personality or other scales without significantly compromising reliability or validity. The short form showed the same pattern of relationships with happiness and environmental variables as the longer scale. Thus, the NR-6 is a reasonable proxy for the 21-item nature relatedness scale where time or space are limited.

### Conflict of interest statement

The authors declare that the research was conducted in the absence of any commercial or financial relationships that could be construed as a potential conflict of interest.

## Footnotes

^1^The wording of nature relatedness items was slightly adjusted to be consistent with the format of the Big Five Factor Inventory. Several foils were added to the measure, as distractors.

## Appendix A

### Short form version of the nature relatedness scale (NR-6)

**Instructions**: For each of the following, please rate the extent to which you agree with each statement, using the scale from 1 to 5 as shown below. Please respond as you really feel, rather than how you think “most people” feel.

**Table:**

**1**	**2**	**3**	**4**	**5**
**Disagree strongly**	**Disagree a little**	**Neither agree or disagree**	**Agree a little**	**Agree strongly**

1. My ideal vacation spot would be a remote, wilderness area.
2. I always think about how my actions affect the environment.
3. My connection to nature and the environment is a part of my spirituality.
4. I take notice of wildlife wherever I am.
5. My relationship to nature is an important part of who I am.
6. I feel very connected to all living things and the earth.

*Scoring Information*: NR-6 score is calculated by averaging all 6 items.

## Appendix B

**Table B1:** **Nature relatedness scales—means and standard deviations**.

	**NR-6 mean (*SD*)**	**NR-21 mean (*SD*)**
Study 1 (*n* = 184)	3.00 (0.86)	3.28 (0.60)
Study 2 (*n* = 145)	3.39 (0.85)	3.67 (0.58)
Study 3 (*n* = 354)	3.34 (0.96)	3.65 (0.68)
Study 4 (*n* = 84) community, baseline	3.44 (1.01)	–
Study 4 (*n* = 123) students, baseline	3.11 (0.96)	–
Study 4 (*n* = 84) community, 1 month	3.56 (0.95)	3.71 (0.67)
Study 4 (*n* = 123) students, 1 month	3.06 (0.91)	3.40 (0.66)
